# Optimizing Academic-Practice Partnerships to Promote Transition to Nursing Practice

**DOI:** 10.1177/08445621251366583

**Published:** 2025-08-20

**Authors:** Kathryn L. Halverson, Michelle Lalonde, Judy Duchscher, Shabneez Xin, Caroline Currie, Andrea Raynak

**Affiliations:** 1 7497 School of Nursing, Lakehead University, Thunder Bay, Ontario, Canada; 2 Department of Nursing, Brock University, St. Catharines, Ontario, Canada; 3 6363 School of Nursing, University of Ottawa, Ottawa, Ontario, Canada; 4 Institut du Savoir Montfort, Montfort Hospital, Ottawa, Ontario; 5 70422 School of Nursing, Thompson Rivers University, Kamloops, British Columbia, Canada; 6 27373 Thunder Bay Regional Health Sciences Centre, Thunder Bay, Ontario, Canada

**Keywords:** nurses as subjects, transition to practice, nurse retention, transitions, uncertainty, workforce issues

## Abstract

**Background:**

An academic-practice partnership was implemented in Northwestern Ontario with the goals of enhancing cross- sector collaboration, co-creating research knowledge related to transition to practice, engaging and recruiting nurses, and mobilizing knowledge to improve the transition experience. There is a growing nursing shortage requiring novel solutions to support retention, particularly for rural and remote populations. Academic-practice partnerships can be leveraged to improve working conditions and consequently job satisfaction (Padilla & Kreider, 2020; Rogers et al., 2020).

**Method:**

Using qualitative methodology, semi-structured virtual interviews were conducted with nine Registered Nurse participants ranging in experience from three to seven months employed at the same hospital in Northwestern Ontario.The interview guide was developed collaboratively by an advisory board comprised of the researcher, hospital staff and input from two student ambassadors from the graduating class. Thematic analysis was completed and broad categories were established with data then expanded into five overarching themes.

**Results:**

Five themes representing impactful sentiments shared by the new graduate nurses were identified: “I couldn’t be the nurse I know I could be”; “I’m with you right now”; “You have to catch up”; “Do you want to learn it with me?”; and “I feel thrown in and unprepared”.

**Conclusion:**

New graduate nurses experience a dissonance between expectations and reality influenced by their interactions with preceptors and colleagues. Academic-practice partnerships can create supportive learning environments, allowing new nurses to transition to independent practitioners while establishing stronger professional identity, which is a positive indicator for retention.

## Introduction

In May of 2023, the Center for Disease Control and Prevention (2023) officially declared the end of the COVID-19 pandemic. The fallout from this global health emergency has paved the way for a new critical concern – a catastrophic shortage of nurses worldwide. There are many contributing factors to the aforementioned shortage that include, but are not limited to; an increase in population size and population aging (Hung & Lam, 2020; Yacuboski, 2019), competing career and family responsibilities (Moloney et al., 2018), mental health challenges (Vahedian-Azimi et al., 2019), experiences of moral distress (Witton et al., 2023), and burnout (Rhéaume et al., 2022). The Registered Nurses Association of Ontario (RNAO) has projected alarming levels of attrition following the pandemic, including Registered Nurses (RNs) aged 26–35 indicating an intention to leave the profession at a rate four times the pre-pandemic rate for that age cohort (Hooper, 2023; RNAO, 2021). The impact of these staffing shortages is not equitably felt, with rural and remote populations experiencing greater challenges in recruitment and retention (Levesque, 2021; Sablik, 2022). Greater distance to amenities, lack of professional growth or career opportunities and a challenge in maintaining professional boundaries due a lack of anonymity were attributed as deterrents to rural employment for healthcare workers (Asghari et al., 2017). Supporting the recruitment and retention of nurses is particularly critical for rural and remote populations, thus novel solutions are required to support nurses in these areas.

A strategy to support nursing workforce stability can be the utilization of academic institution and practice setting partnerships. Halili et al. (2022) define academic-practice partnerships as strategic relationships between an educational institution and a practice setting. Within nursing, these partnerships involve joint ventures to achieve improved outcomes in education through clinical placements, shared research initiatives and evidence-based practice to improve patient care (Halili et al., 2022). The partnerships may form with varying degrees of structure, but often require multi-disciplinary and multi-level cooperation, from clinical nurses and nursing students to management and faculty (Halili et al., 2024). Academic-practice partnerships are mutually beneficial relationships designed to facilitate collaboration and benefit for both parties (Bacon & Jenkins, 2023). The academic partner offers research expertise while the clinical partner facilitates data collection and lends context to data interpretation within a practice environment (Bacon & Jenkins, 2023). Such partnerships are also pragmatic for the integration of evidence-based practice, allowing the facilitation of knowledge dissemination to influence the delivery of nursing care (Halili et al., 2024). Academic-practice partnerships nurture nursing education through shared values of teaching and scholarly advancement, ultimately benefiting the emerging needs of the patient population whilst simultaneously improving recruitment and retention through improved working conditions (Bejster et al., 2022).

Pearson et al. (2015) suggest that nurses working within academic-practice partnerships report higher satisfaction in their interactions with students in clinical placements, better engagement with clinical activities, and personal motivation to pursue higher education. The implementation of such partnerships has considerable value for practice settings, with research demonstrating that precepted staff report a higher sense of fulfillment and improved retention rates for both preceptors and their students (Padilla & Kreider, 2020; Pearson et al., 2015; Rogers et al., 2020). Consequently, nurses that demonstrate higher levels of job satisfaction demonstrate a stronger sense of professional identity (Fitzgerald, 2020). Academic-practice partnerships have the transformative potential to strengthen the nursing profession through the improvement of the transition to practice experience for new graduate nurses (NGNs).

## Study Aims & Objectives

This article presents the results of a study conducted to address an urgent need to recruit and retain nurses and other health care professionals in the predominantly rural region of Northwestern Ontario. Working in partnership with a university and hospital partner, the aims of this study are to: (a) examine factors influencing the recruitment and retention of new nurses; (b) improve the understanding of these factors and use the knowledge to guide the practice partner to design and implement processes that support and influence recruitment and transition to practice; (c) facilitate collaboration between the academic-practice partners to support scholarly inquiry regarding the transition to practice experience; and (d) foster an opportunity to evaluate and improve the quality of the transition experience through knowledge mobilization initiatives that facilitate the flow and exchange of research knowledge across sectors. The objectives of this study are to: collaborate, engage and recruit, evaluate and mobilize knowledge with a focus on quality improvement. The research question guiding this study is: How can academic-practice partnerships support recruitment and retention of new graduate nurses?

## Background & Significance

With a current and pressing nursing shortage, the need to recruit, educate, retain, and support new nurses is more important than ever before. According to the Canadian Union for Public Employees (CUPE), due to high turnover, vacancy rates for nurses in inpatient settings have exponentially increased in the last eight years, measuring 300% higher in 2023 than in 2015 (2023). The RNAO projects an alarming exodus from the profession following the pandemic as a result of strained mental health and well-being (RNAO, 2021).

Nursing students are not immune to the impact of the stressful environments in which they receive their training. Burnout amongst nursing students which has already been assessed as higher than students in other disciplines, has steadily increased and may contribute to higher rates of attrition (Burleson et al., 2023). Attrition disproportionately occurs within the nursing student population, with medical students less likely to leave their programs before completion when compared to midwifery or nursing students, related to burnout attributed to negative interactions with preceptors, emotional strain and poor support for mentally distressing situations (Neiterman et al., 2023). Reported statistics for attrition vary internationally due to a lack of standardized reporting metrics (Canzan et al., 2022). Countries such as the United States, Australia and Canada report ranges of attrition from 10–50% while England reports a range of 25–40% (Canzan et al., 2022).

Nursing students indicate a dissonance between expectations and reality, with the clinical environment experienced as faster paced and more diverse than they anticipated (Ching et al., 2020). This dissonance is pervasive into the transition period for the NGN, where the level of support and supervision diminishes, while confidence and proficiency are expected to increase. The resulting “reality shock” influences professional identity formation and ultimately impacts retention (Kim, 2020; Sargis & Kramer, 1975). Students report feelings of incompetence and note that their learning is often deprioritized compared to the regular responsibilities of patient care (Ching et al., 2020). Nursing students should not be considered an extension of the hospital workforce, nor a solution to staffing shortages as this results in compromises in their educational opportunities. Similarly, NGNs should be afforded the opportunity to adapt and transition without the pressure or expectation to practice with the proficiency of a seasoned nurse.

Research that involves partnerships and collaboration between education and practice are considered critical in the effort to bridge the academic-practice gap (Huston et al., 2018). Nurse employers are well-positioned to consider the impact of factors that influence transition to practice and professional identity formation, as well as to promote job satisfaction, foster a culture of respect, and retain new graduates. A study conducted by Spector (2015) on transition to practice found a need for ongoing support of the new graduate during the entire first year of practice, with the first six- to nine-month period seeming to be the most vulnerable time. Academic institutions and practice settings have an opportunity and obligation to support nurse transition by communicating realistic expectations and designing opportunities for role experimentation and implementation (Halverson et al., 2022).

The Royal Society of Canada issued a report in 2022 titled *Investing in Canada's Nursing Workforce Post-Pandemic: A Call to Action*. This report highlights four recommendations, including the strengthening of the voices of nurses in policy and planning at multiple levels to promote the valuation of the nursing workforce (RSC, 2022). In addition, the Government of Canada's *Health Human Resources Symposium Summary Report* suggests a short-term action to create positive workplace culture and practice environments is to assess the current health workforce culture through focused engagement (i.e., interviews) with frontline healthcare workers (2022). For these reasons, this study was designed around a qualitative approach to capture the experiences of NGNs through focused interviews, thus strengthening their voices and providing valuable insight into the transition experience.

## Theoretical Underpinnings and Methodological Support

Benner's Novice to Expert model (1984), Duchscher's Stages of Transition Theory (2008), and Johnson et al.'s Professional Identity Pathway (2012), offer theoretical and methodological support for the study.

Benner's early work is based on stages of clinical competence, suggesting evolution through the following stages: novice, advanced beginner, competent, proficient, and expert (Benner, 1984). Later work expanded on this theoretical perspective to highlight the value of academic and practice partnerships and collaboration. This study builds on the literature suggesting academia and practice collaborate to navigate and inform the shift from socialization to personal and professional transformation that has been encouraged in nursing education (Benner et al., 2010; Brykczynski, 2014; Gilmore, 2014). In bridging the academic-practice gap with partnerships such as this, we can coach students/professionals to recognize the bigger picture and what is most important (Benner et al., 2010). This study bridged the academic-practice gap by designing and implementing a fully collaborative research study led by an advisory board comprised of 12 nurse educators employed by the hospital, hospital administration, nursing faculty, and student ambassadors. The advisory group met monthly for a seven-month period.

NGNs have been found to evolve through three stages identified as doing, being, and knowing during their first 12 months of practice (Duchscher, 2008). While each NGN experiences a unique transition experience, the first 12 months of employment encompass a relatively consistent pattern of emotional, intellectual, physical, sociocultural, and developmental issues (Duchscher, 2009). According to Duchscher (2012), progress through the stages of transition is influenced by length and quality of orientation and the transition and integration programs graduates are enrolled in upon hiring. The program outlined in this study was designed to supplement the length and quality of the pre-existing orientation at the hospital partner site, an enhancement expected to help attract and engage graduating students. Duchscher's (2009) finding that each nurse experiences a unique transition has informed the decision to complete individual interviews and also to direct graduating students to the Nursing the Future™ website to access resources and support as they prepare for this transition.

One subsection of the professional identity pathway (Johnson et al., 2012) titled “professional identity and transition to practice” focuses on the dissonance between expectations and experiences and how this poses a threat to retention. Understanding dissonance between expectations and experiences helps us to appreciate factors and events that may threaten retention (Halverson, 2020). The theoretical perspectives of Benner (1984), Duchscher (2008), Johnson et al. (2012) and the findings of prior research conducted by Halverson (2020) have informed the objectives and methods of this study.

## Methods

### Research Design

This study utilized a qualitative description design to remain closely aligned with the real world setting and experiences shared by the participants within the phenomena of inquiry (Bradshaw et al., 2017). Beginning with a literal description, the researchers interpreted findings through the lens of a theoretical framework, avoiding abstraction from what was shared (Bradshaw et al., 2017). Duchscher's (2009) Theory of Transition Shock was used as the overarching framework for this study. Philosophically, qualitative description honours relativism, acknowledging that realities are multiple and subjective (Bradshaw et al., 2017). The research team ensured that experiences were represented from the perspectives of the participants, to provide a rich description and an emic view of the subject matter (Bradshaw et al., 2017).

### Context

The hospital partner is the primary acute care facility for the region of Northwestern Ontario, serving a population of over 250,000 residents dispersed over a large, primarily rural, geographical area and reporting unsustainable nursing vacancy rates at the institutional level. The practice partner is a 375-bed teaching hospital engaged in academic partnerships with the host university and other educational institutions, supporting clinical placement needs of over 1,200 students per year from over 25 disciplines varying from nursing to medical radiation sciences. At the time of the research project, the goals aligned with the hospital partner's long-term strategic priorities by building on academics, improving the capacity of staff, and teaching the next generation of health care professionals while also fulfilling the host university's strategic goal of partnership and collaboration.

### Data Generation

Using qualitative methodology, semi-structured interviews were conducted with nine participants and aimed to capture the unique experiences of new nurses. Most interviews were conducted virtually with two of the interviews completed in-person at the academic institution. Interviews were conducted by the primary investigator, a nurse researcher, employed by the academic partner. The primary investigator led the research team for this study and had previously occupied the role of professor for some participants during their baccalaureate education.

The interview guide was developed collaboratively by the advisory board comprised of the researcher and hospital staff with input from two student ambassadors from the graduating class. Examples of lines of inquiry included in the interview guide include: “Can you tell me about how becoming a nurse is or is not what you expected it to be?”; “Do you see yourself continuing to work in acute care? Why or why not?”; “In what ways do you feel supported?”; “Are there opportunities for you to feel more supported?”; How do you feel about the nursing profession as a whole?”; “How do you feel about your future career as a nurse?”. The duration of each interview ranged from 30 min to two hours, with most interviews lasting approximately 40 min. The data was professionally transcribed.

### Sample and Recruitment

The sample included seven female and two male Registered Nurses, all 20–30 years of age. Participants ranged in experience from three to seven months and were employed in acute in-patient care areas including emergency, intensive care, neonatal intensive care, medical, and surgical areas caring for a variety of patient populations. All NGNs were within their first year of practice to gain a better understanding of their experiences during the period of time Duchscher (2009) describes as most challenging in terms of transition. Participants were recruited via email invitation with correspondence sent by the hospital partner to the new nurses hired in Spring 2022. Consistent with qualitative research methodologies, the smaller sample size was anticipated and considered sufficient to address the research question and aims of the study based on the data generated from the interviews (Bradshaw et al., 2017).

### Data Analysis

Thematic analysis was chosen for this project because of its flexibility, usefulness in examining unique perspectives of different participants, and potential for revealing unanticipated insights in the data (Braun & Clarke, 2006; Nowell et al., 2017). Data analysis was executed manually and systematically documented in Microsoft Word, with files disseminated and exchanged among research team members as required. The primary investigator who conducted the interviews and a Masters-prepared research assistant with experience in thematic analysis completed an initial reading of the transcripts focused on familiarization with the data while ascertaining narratives embedded in the experiences of new graduate nurses. These initial readings generated codes which were then categorized to define and name themes through decisions that were tracked through analytic memos, where the research team documented thoughts related to the data such as reflections, conclusions, and connected ideas. This documentation served to generate an audit trail to track the inductive analysis process (Braun & Clarke, 2006; Saldaña, 2020). Subsequently, broad categories that were verified by two readers were established and consensus was reached. This stage allowed researchers to become intimately familiar with the experiences of each NGN (Sandelowski, 1995). These broad categories were then collapsed, or in some cases, expanded, into five major themes, which were verified through returning to text exemplars. During this stage, conclusions were held lightly as recommended by Miles and Huberman (1994), allowing for flexibility in changing themes and groupings as new ideas emerged. Finally, definitions for each theme were established and the title of each was refined. The flexibility of thematic analysis allowed for the extraction of themes that resonated with the research objectives while accurately reflecting the perspectives of the NGNs (Cernasev & Axon, 2023). Dominant themes emerging from the interviews are outlined in the Findings section and Table 1.

**Table 1.** Themes and Participant Quotations.

Theme	Quotations from participants
“I couldn’t be the nurse I know I could be”	*“About three days after my orientation, having 6–10 patients on day shift, I started not loving my job anymore. I couldn’t be the nurse I know I could be. I started not being the professional that I know I can be.” (Participant “Kallie”)* “*I didn’t expect it to be as scary as it was. I come in early to research everything because it takes one wrong move to potentially kill someone. These patient's lives are in your hands. You can’t control if you have three patients or nine patients on a day shift.” (Participant “Darius”)*
“I’m with you right now.”	*“I had a patient who was very anxious and I just held her hand for a second and she said ‘I know you have so many other patients’ and I said, ‘I have a lot of patients but I’m with you right now.’ And spending three minutes with her, it was enough to turn her day around. Those are the things that I like.” (Participant “Daphne”)* *“You can be fully staffed and only have three or four patients. In that case you’re able to provide full care and give full showers and baths and shaves and do everything you can to help those patients.” (Participant “Darius”)*
“You have to catch up.”	*“I’ve been given a lot of freedom to provide care. But I don’t know if that's because I’m allowed or because of the lack of overhead. Who knows? Maybe I’m providing inappropriate care. I can’t tell because I don’t feel there is enough supervision.” (Participant “Darius”)* “*My preceptor saw that I was stressed and struggling and just took over for me. But that way, I didn’t have the chance to explain my thought processes. It was a little hurtful.” (Participant “Shadow”)*
“Do you want to learn it with me?”	*“I get to sit down with the NRT managers and discuss my strengths and weaknesses. That was very helpful.” (Participant “Shadow”)* “*My preceptor would say, ‘so let's say this situation is happening, how would you draw up this medication?’ Or, ‘the doctor asks you to get ready to perform this procedure, how would you do that?’” (Participant “Sophie”)* “*Staff ask: ‘is this a new skill? Do you want to go and learn it with me?’ They’re really good at teaching.” (Participant “John”)* “*Sometimes on night shifts there are not resources in place to help junior staff. For example, one night we had a patient who was ordered fibrinogen and nobody on our floor had given that before and we couldn’t find a policy on it. We found supplies to use but we did not know how to run it correctly. We called oncology and the ICU for help but they were not entirely sure. We did not have a clinical nurse specialist on, so we found a policy online from another hospital in Ontario, on how to infuse fibrinogen.” (Participant “John”)*
“I feel thrown in and unprepared.”	*“Being told ‘you need to read these policies,’ sometimes it just goes in one ear and out the other, despite your wanting to learn. Reading how to draw up a medication is so different than actually doing it.” (Participant “Sophie”)* “*In medical school, you are a resident for at least two years, sometimes up to seven depending on the specialty. I don’t understand why nursing is so different. I think having more specialized clinical training during nursing school would be really nice.” (Participant “Sophie”)* “*I feel I was supported during my orientation but afterwards, I was on my own. I feel like asking questions to senior nurses sometimes becomes annoying to them. That feels discouraging. I see a lot of diagnoses, etc. now that I did not see while doing my orientation.” (Participant “Marie”)* “*I had a patient who was hypoglycemic. I’ve never had to follow the hypoglycemia protocol before. The nurse working with me was just telling me what to do but it was not helpful. She said ‘why hasn’t the IV been started? Why haven’t the bags been primed?’ Meanwhile, I said ‘I’m still reading the protocol, I have no idea what I’m supposed to be doing.’” (Participant “Marie”)*

*Note*: Direct quotations from study participants organized into dominant themes.

## Ethical Considerations

Ethics clearance was provided by Lakehead University's Research Ethics Board (File#1469196) and Institutional Authorization (RP-802) was granted from the hospital partner. Participants completed a written informed consent prior to participating in an interview and confidentiality and privacy protocols with relation to data storage were shared. Participants selected a pseudonym. All forms and electronic data were securely stored on password-encrypted devices. Consent forms and transcripts were stored separately to protect the identities of participants. Copies of files and transcripts were deleted from the devices of the research team at the conclusion of the study.

## Findings

Five themes were identified, evolving from the impactful sentiments shared by the NGNs. See Table 1 for additional quotations from study participants.

### “I Couldn’t Be the Nurse I Know I Could Be”

Capturing the frustration, disappointment and isolation expressed by the NGNs, the broad category of dissonance between expectations and reality was identified. This was further isolated into the theme of “*I couldn’t be the nurse I knew I could be,”* reflecting the strain of performing below the expectations set by one's own self. The NGNs interviewed shared experiences of significant dissonance between the idealized practice they envisioned while in school, compared to the reality of the healthcare environment. The nurses felt unable to deliver care plans as intended due to the overwhelming nature of their patient assignments. The reduction of holistic care into task-based work left the NGNs feeling defeated and detached from the essence of what they understood nursing to be, that is, a profession of caring. The NGNs described feeling that the support and supervision they received as nursing students was unavailable to them now, contributing to a sense of inadequacy. One participant stated:

“When I was a student on that floor, I loved it. I was so excited to start and be a nurse. I couldn’t wait. I felt so prepared. I was ready to be on my own. Now, I feel like I’m back in first year nursing, no idea what I’m doing … In the moment, it's so much different than being a student, having that continuous support with you.” (Participant “Kallie”)

This participant's experience of excitement and pride at their achievement of becoming a nurse was eroded as they practiced as a new RN. Despite completing an intensive program with academic and practical hours, this NGN reflects on feeling like a novice, just as they had when they began their nursing education.

### “I’m With You Right Now”

The rewarding nature of therapeutic relationships was another broad category identified within the experiences of the NGNs. Moments of authenticity and connection where the NGNs felt they were able to impact patients and be present was reflected in the theme of “*I’m with you right now.*” The NGNs reflected upon positive patient encounters where they were able to deliver relational practice, which they associated with higher quality care. Adequate staffing levels were identified as a substantial factor in the NGNs being able to connect with their patients and recognize the social determinants that may have influenced their current hospitalization. One NGN recalled a conversation with a patient where they stated:

“I had a few minutes here and there and I spent some time with my patient and he would sing songs to me. I was talking to him and asking ‘how did you get here? If you go home, do you have any supports so that we can fix this and you’re not going to be back here? And are you ready to make changes in your life?’” (Participant “Daphne”)

With additional time in their day, this NGN was able to foster a therapeutic relationship with their patient and expand their clinical assessment by viewing the patient with a holistic lens. Meaningful interactions made an impact on both the NGNs and their patients as the nurses described feeling fulfilled and valued in their rewarding work.

### “You Have to Catch Up”

The broad category of negative experiences with colleagues was identified in the reflections shared by the NGNs. To encompass the sense of urgency, inadequacy and hostility, the theme of “*You have to catch up”* emerged from the data. As NGNs, the participants expressed concerns with the level of oversight available during their transition to practice. The NGNs describe feeling insecure in their practice as they lacked the appropriate supervision to validate their actions. Negative interactions and condescension led to the erosion of learning opportunities with preceptors completing the tasks themselves, rather than taking the time to demonstrate and teach the NGNs. One participant recalled such an instance:

“I explained to my preceptor that it had been a while since I had done bedside nursing. I asked her to be patient with me but she was hostile. She said ‘we can only do this in one day and you have to catch up.’” (Participant “Shadow”)

The experience of this NGN illustrates challenges of the orientation process, where the limitation of time and support leads to unrealistic performance expectations. The NGNs felt unprepared for autonomous practice, magnified by inadequate learning experiences with their preceptors.

### “Do You Want to Learn it with Me?”

Contrastingly, the NGNs were also able to acknowledge the positive experiences they had with their colleagues as impactful during their orientation. This category was reflected within the theme of “*Do you want to learn it with me?”,* embodying a sense of tenderness and collegiality between an NGN and their preceptor. The NGNs shared that positive encounters with staff and management facilitated their transition to practice. Senior staff on the units would assist with situations they had not encountered before or run through hypothetical situations to support the development of their critical thinking. The NGNs recalled problem solving and collaborative efforts to provide competent care with limited clinical resources on night shifts. Notably, the NGNs described the benefits of having a designated resource staff member per shift that was available for questions beyond their initial six-week orientation period. One NGN explained this as:

“My supervisor said that she would assign me a go-to nurse in case anyone's busy. I think that's helpful with transitioning from being on orientation, to working independently.” (Participant “Shadow”)

Another NGN shared how having an opportunity to reflect upon their strengths and weaknesses with managers on a regular basis was helpful as they progressed through their transition to practice.

### “I Feel Thrown in and Unprepared”

Reflecting upon their orientation experiences, the NGNs were able to identify challenges they encountered transitioning from supported students to independent nurses. The broad category of learning needs was embodied by the theme of “*I feel thrown in and unprepared”* to capture the underlying desire to enact changes to education, orientation and better support future nurses. The NGN discussed the challenges in bridging their academic education with the clinical realities in which they were expected to provide nursing care. The NGNs contrasted their limited training with the extensive residencies provided to medical students, lamenting that a handful of precepted shifts does not provide sufficient exposure to diverse patient situations. One NGN felt this was particularly true for specialized units, stating:

“Orientations should be longer. I don’t think someone is qualified to be working in an intensive care unit after four weeks. I extended my orientation by two days which was a little helpful but in the grand scheme of things, what will I learn in an extra two shifts that I didn’t already know?” (Participant “Marie”)

Ensuring all potential patient situations are explored during orientation would not be feasible, so the NGNs suggested a variety of teaching methods may be more conducive to their learning, rather than simply reading institutional policies and standards. Nursing education is comprised of theory, lab sessions and hands-on practical hours. Similarly, new graduate orientation may include a combination of methods to deliver necessary information. One NGN reflects:

“I work in a specialized area as a new graduate nurse and we did not have exposure in nursing school to many things that pertain to my unit. Without theory and lab orientation components, I feel thrown in and unprepared.” (Participant “Sophie”)

Simulating patient situations in a safe training environment allows nurses the opportunity to rehearse potential actions, mitigating some of the uncertainty an NGN may face when practicing independently. The NGNs were cognizant of feeling like a burden to senior staff with questions that persisted beyond their initial orientation period. Unknown situations served as a significant source of stress, as the NGNs struggled to collaborate with senior staff who might dictate actions to be completed, rather than support problem solving. Enhancing orientation for NGNs may provide greater support during the transition to independent practice.

## Discussion

**Figure 1.**
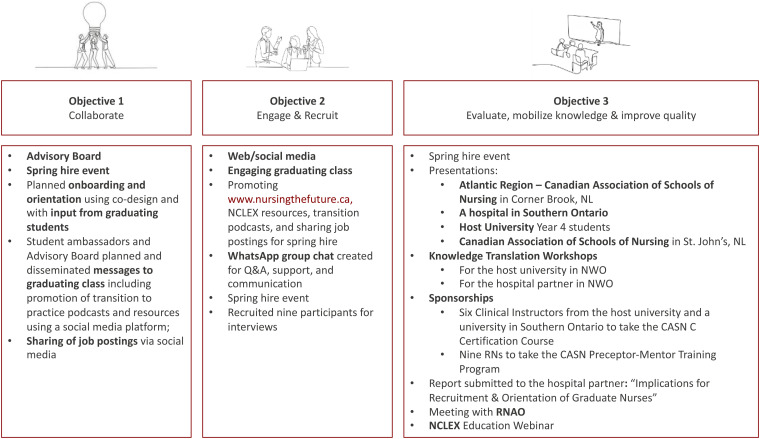
Study Objectives and Knowledge Mobilization Activities. *Note:* Chart Detailing the Primary Study Objectives with Corresponding Knowledge Mobilization Activities.

The stories shared by the NGNs illustrated tensions experienced during the progression of their independence as nascent health professionals with implications for overall recruitment and retention. See Figure 1 for a fulsome list of knowledge mobilization activities organized by study objective.

### Collaborate

Nursing students and employed RNs are at risk of similar occupational health related stressors of uncertainty, heavy workloads and compassion fatigue (Ching et al., 2020). Canadian nursing education is typically comprised of two to four years of undergraduate study at a college or university level with varied clinical placements in primary care, mental health, community-based settings, obstetrics in addition to inpatient hospital sites. Despite exposure to clinical situations throughout their nursing training, new graduates are at risk of transition shock as the supportive network of peers and preceptors they grew accustomed to as students are left behind (Duchscher, 2009).

Erdat et al. (2024) describe several structural considerations that impact the experience of transition shock in NGNs, including difficulties applying learned theory to clinical practice, strained interdisciplinary communication, and time management. Burdened by high workloads and patient acuity, NGNs are consequently more likely to miss critical components of holistic nursing care such as patient education, communication and offering emotional support (Erdat et al., 2024; Zhao et al., 2024). With transition shock impacting holistic patient care, recognition and intervention to support NGNs is critical. The nurses interviewed in this study shared such experiences of dissonance as they felt unable to practice to the standards they had internally set while progressing through their nursing education.

This discrepancy between education and practice results in reality shock, which also impacts professional identity formation and was captured within the theme “*I couldn’t be the nurse I knew I could be”* (Kim, 2020; Sargis & Kramer, 1975; Zhao et al., 2024). This reflects the tension between the idealized professional, shaped during nursing education and the reality of practice underscoring the struggle that new nurses experience reconciling their professional identity with the realities of the healthcare system. Practice partners must facilitate both clinical skill development and socialization into the professional role.

In this study, the development of an advisory board allowed peer support networks to disseminate valuable information regarding the credentialling process, employment opportunities, application processes, and onboarding. Such strategies not only support recruitment but stimulate conversations that promote coping skills to mitigate academic stressors (Nordstrom et al., 2018). A study conducted by Tousignant et al. (2021) further reinforces the strengths of peer relationships by suggesting that the use of student pairs or dyads, may improve learning by way of enhanced confidence and competence. The practical significance of such collaboration is captured within the theme of “*Do you want to learn it with me?”* where the NGNs reflected upon constructive preceptor experiences. Similar to the mutual advantages found for academic-practice partnerships, nurse preceptors and student or NGN mentees can both benefit from a positive preceptorship experience. While the student seeks learning opportunities to develop clinical experience, the preceptor reflects upon their own assessments and nursing care to strengthen decision-making (Flott et al., 2022).

### Engage and Recruit

New graduate RNs experience a volatile period of transformation as they evolve from students into independent practitioners. Engaging nurses who have left this vulnerable period provides valuable insights and quality improvement opportunities for nursing education and orientation at hospital partner sites. The nurses interviewed in this study shared experiences where they felt devalued as learners or had inadequate supervision to validate their practice. The hospital partner's strategy of assigning a resource nurse to an orienting new hire is aligned with the recommendations of Duchscher (2008), with current evidence also suggesting that access to a senior nurse is considered vital when new skills or experiences are encountered in acute care settings (Smith et al., 2021). Benner's (1984) proposition that mentorship facilitates NGN transition was reflected in these findings, with participants expressing a strong desire for structured mentorship and guidance from experienced nurses to help bridge the gap between academic preparation and clinical reality. NGNs emphasize the significance of support, encouragement and feedback from preceptors as they transition and develop confidence, particularly in their first year of practice (Charette et al., 2023; Smith et al., 2021). The use of a messaging group chat provided a platform to share constructive resources with the graduating class of the host university and offer an opportunity for authentic presence, capturing the essence of the theme of “*I’m with you right now”*. Students who have been mentored by alumni report greater access to learning experiences and an enhanced awareness of the expectations of the nursing profession (Allred & Sakowicz, 2019). An additional benefit is the generativity and sense of satisfaction experienced by alumni mentors (Allred & Sakowicz, 2019).

Academic-practice partnerships can serve the hospital partner through opportunities for recruitment during clinical placements. This is particularly true of specialty areas, where NGNs may be considered ineligible due to inexperience (Manz et al., 2021). According to Manz et al. (2021) students who participated in a perioperative preceptorship in their final term reported a greater understanding of the perioperative nurse role, improved time management and interdisciplinary collaboration, leading to enhanced patient safety.

Similar programming may be adapted to promote workforce diversity, as demonstrated by a study conducted by Johnson et al. (2024), which examined how immersion internships at historically Black colleges and universities could promote recruitment for nurse practitioner programs. Follow up with participants indicated ongoing engagement with facilitators of the immersion program, with many seeking letters of recommendation to pursue further advanced practice nurse training and professional roles (Johnson et al., 2024). With an estimated 7% of nurse practitioners identifying as Black in the United States, academic-practice partnerships may be used to mitigate disparities in opportunities and enhance representation of diverse groups (Johnson et al., 2024).

### Evaluate, Mobilize Knowledge and Improve Quality

Themes within the NGNs narratives of “*You have to catch up”* and “*I feel thrown in and unprepared”* highlight instances that reveal the often-disjointed nature of the transition to practice. Preceptors are an integral part of a nurse's transition experience not only for the demonstration of clinical skills, but the essential socialization and workforce integration they support (Hong & Yoon, 2021; Lalonde & McGillis Hall, 2017). There remains a critical gap in how undergraduate nursing education prepare graduates for the realities of clinical practice. This resonates with Benner's (1984) theory, where the transition from novice to competent nurse is seen as a developmental process requiring not only technical skill acquisition but also the maturation of clinical judgment and decision-making.

Organizational frameworks and institutional policies may facilitate or hinder the workforce integration of NGNs. NGNs in this study frequently reported feelings of isolation and inadequate supervision, which can be attributed to structural deficiencies such as understaffing, inadequate orientation programs, and unclear role definitions. These systemic issues are consistent with findings from Johnson et al. (2012), which propose that inadequate institutional support significantly contributes to attrition and impacts professional identity formation among NGNs.

While attrition rates vary both locally and globally, studies suggest the intent to leave within the first 12 months of practice for an NGN may exceed 40% (Hong & Yoon, 2021). This is correlated with poor preceptorship, impacting the nurse's socialization to the profession at large (Hong & Yoon, 2021). Training clinical instructors and preceptors can offer a positive experience for nursing students and new graduates, additionally influencing retention (Hong & Yoon, 2021). Interpersonal skills and emotional intelligence can foster the evolution of teachers that are better suited to supporting new graduates in navigating the tumultuous transition period (Crawford et al., 2019; Lalonde & McGillis Hall, 2017).

The participants interviewed shared experiences which resonated with incivility amongst nurses. Studies indicate incivility has remained prevalent at consistent levels amongst nurses over the last twenty years (Crawford et al., 2019). The implementation of preceptor and clinical instructor programs can better prepare teachers to recognize such instances as they occur and prevent a culture of bullying from becoming innate. When describing traits that an effective preceptor may possess, students identify key concepts such as motivation, dedication, kindness, approachability and deriving inherent pleasure from their work (Manuel Martinez-Linares et al., 2019). These traits are congruent with nurses that have an established sense of professional identity (Halverson, 2020). Mobilizing clinical instructors and preceptors that have a strong sense of professional identity provides a source of aspiration for novice nurses (Halverson, 2020).

In the initial stages of their profession, new nurses are focused on cementing their understanding of clinical skills, with a lower priority on relational practice (Halverson, 2020). The participants interviewed for this study shared experiences of therapeutic relationships as memorable and most fulfilling in their practice. Supporting a successful transition to practice will create nurses who are better able to meet the complex intersectional needs of their patients, as they have been trained by nurses who have modelled the same behaviour.

To further explore these ideas, future research could benefit from a more comprehensive examination of the organizational structures that support or impede the successful integration of NGNs. Longitudinal studies that track NGNs over time and across different practice settings may provide valuable insights into how institutional practices shape the transition process.

## Strengths and Limitations

Data analysis was completed by two research assistants, thus mitigating the opportunity for researcher bias, specifically confirmation bias which may have contributed to the primary researcher interpreting the data in a way that supported their hypotheses. Participants represented multiple clinical areas in the hospital and thus had varied experiences with onboarding (different durations for orientation and mentorship prior to independent practice) and factors such as supervision and support, patient acuity, scope of practice, workload, and patient ratios.

This study is limited in that the participants were all employed by one organization by nature of the partnership, funding arrangement, and the local context. This limits the interpretation of the findings as all participants worked in an acute care inpatient care setting, thus not inclusive of the transition experiences of nurses working in other practice areas such as community health. By nature of the study design, only one interview was conducted with each participant, thus limiting the opportunity for insight into their dynamic and evolving transition to practice. Future research might aim to utilize longitudinal approaches to capture the transition as it is experienced over time (for example from student to one year into practice). Interviews were conducted by the primary investigator who was employed by the academic partner and had previously known some of the nurse participants in their student capacity, thus possibly contributing to participant bias. A strength of this approach is that the interviews were not conducted by the employer, which may have helped the participants to openly share their experiences. This arrangement highlights a strength of approaching transition to practice research with new nurses in the context of an academic-practice partnership.

## Conclusion

The aim of this endeavor was to facilitate collaboration between the academic-practice partners to support scholarly inquiry regarding the transition to practice experience with implications for recruitment and retention. Program objectives fostered an opportunity to evaluate and improve the quality of the transition experience through knowledge mobilization initiatives that facilitated the flow and exchange of research knowledge across sectors. The implications of this study have potential for informing the design and implementation of academic-practice partnerships focused on understanding and supporting transition to practice in other unique jurisdictions and for other disciplines beyond nursing. There is a need to explore collaborative, cross-sectoral and interdisciplinary approaches that better understand factors influencing recruitment and retention of new nurses in settings beyond the acute care sector, and over a longer timeline to explore the evolution of threats to retention over time. Academia and practice settings have an opportunity to create a culture of collaboration that will support nursing students through their transition to nursing practice in a way that contributes to the recruitment and retention of NGNs.
